# The Fatalistic Decision Maker: Time Perspective, Working Memory, and Older Adults’ Decision-Making Competence

**DOI:** 10.3389/fpsyg.2019.02038

**Published:** 2019-09-12

**Authors:** Michael Rönnlund, Fabio Del Missier, Timo Mäntylä, Maria Grazia Carelli

**Affiliations:** ^1^Department of Psychology, Faculty of Social Sciences, Umeå University, Umeå, Sweden; ^2^Department of Life Sciences, University of Trieste, Trieste, Italy; ^3^Department of Psychology, Faculty of Social Sciences, Stockholm University, Stockholm, Sweden

**Keywords:** time perspective, decision-making competence, decision making, working memory, older adults

## Abstract

Prior research indicates that time perspective (TP; views of past, present, and future) is related to decision-making style. By contrast, no prior study considered relations between TP and decision-making competence. We therefore investigated associations between dimensions of the Swedish Zimbardo Time Perspective Inventory (S-ZTPI) and performance on the Adult Decision-Making Competence (A-DMC) battery in a sample of older adults (60–90 years, *N* = 346). A structural equation model involving four A-DMC components as indicators of a general DMC factor and the six TP dimensions as the predictors revealed a significant negative association between the Present Fatalistic dimension and DMC. Given that age-related differences were apparent in DMC and that Present Fatalistic orientation increased with age, we tested a model by which the age-related differences in DMC were mediated by age-related differences in Present Fatalistic attitudes and in working memory. The results were consistent with full mediation of the age effects, with Present Fatalistic and working memory jointly accounting for a substantial amount of the variance in DMC (51%). The finding that DMC among older adults, in particular more cognitively demanding aspects such as applying decision rules, can be undermined by increased present fatalistic attitudes and declines in working memory is discussed in terms of theoretical frameworks highlighting the contribution of both motivational and cognitive factors to effective decision making.

## Introduction

Recent decades have seen an increased interest in individual differences in decision making and in the individual features contributing to better decision outcomes (see, e.g., Stanovich and West, 1998; Bruine de Bruine et al., 2007). Older adults represent a group of particular interest in this regard, motivated by global population aging (Kinsella and He, 2008). Reviews of the literature and theoretical models (Strough et al., 2015a, b) suggest that older adults’ motivation to put effort into the decision-making process changes with age and depends on several factors, including perceived personal relevance of decisions, maintenance of positive emotions growing more relevant with age (Carstensen, 2006), and confidence in applying ability and knowledge. This holds beyond the ascertained role of cognitive abilities like memory skills and fluid reasoning (e.g., Bruine de Bruin et al., 2012; Del Missier et al., 2015).

Two different aspects of decision making need to be distinguished: decision-making competence and decision-making style (Bruine de Bruine et al., 2007). Decision-making competence refers to effective decision making as reflected by performance measures of accuracy and consistency of the decisions, evaluated in relation to normative criteria (see also Parker and Fischhoff, 2005). Decision-making styles, by contrast, refer to habitual approaches to and handling of situations that involve decision making. Measures from the two domains showed some significant intercorrelations (Bavol’ár and Orosová, 2015), but were also shown to account for unique variance in decision-making outcomes (Bruine de Bruine et al., 2007). Importantly, measures of both competence and style have been found to be related to indicators of real-life decision-making success (Bruine de Bruine et al., 2007; Dewberry et al., 2013; Wood and Highhouse, 2014).

A significant stream of research on individual differences in DMC has risen from the development of performance-based measurement instruments (e.g., Bruine de Bruine et al., 2007; Finucane and Gullion, 2010; Parker and Fischhoff, 2005). These studies showed that individual differences in DMC are stable over time (Parker et al., 2018) and related to individual differences in fluid and crystallized cognitive abilities, although the strength of these relations depends on the type of DMC tasks considered (Del Missier et al., 2012, 2013) and DMC tasks more related to fluid abilities, working memory, and executive functioning seem to show a greater age-related decline (Bruine de Bruin et al., 2012; Del Missier et al., 2012, 2017; for reviews, see also Del Missier et al., 2015; Strough et al., 2015b). As concerns the relation between individual differences in DMC and indicators of real-life decision-making success, some studies showed significant associations between DMC scores and reported negative life decision outcomes (Bruine de Bruine et al., 2007; Parker et al., 2015) or risky and antisocial behaviors (Parker et al., 2018), with higher DMC scores being associated with better life decision outcomes and more constructive behavior.

The aim of this study was to examine older adults’ decision-making competence in relation to a cognitive factor deemed to be particularly crucial to efficient DMC performance in more demanding tasks (Del Missier et al., 2015), namely, working memory (i.e., the capacity for simultaneous processing and storage of information in memory) and the individual’s habitual view of the past, present, and future, known as time perspective (TP). TP has been conceived as a semi-conscious process in which the three temporal frames (past, present, and future) play a central role in the relationship between personal and social experiences (Zimbardo and Boyd, 1999). A basic assumption of this framework is that temporal biases in the form of an over-focus on, or overly aversive attitude toward, some of the frames and attitudes (e.g., a negative attitude toward the past) develop habitually. Once these temporal attitudes have developed, they act as dispositional factors predictive of how an individual will respond across a variety of daily life behaviors and choices (i.e., a trait; recent frameworks making a distinction between TP as a trait and state; Stolarski et al., 2018).

To operationalize TP, Zimbardo and Boyd (1999) developed the Zimbardo Time Perspective Inventory (ZTPI). ZTPI comprises five subscales, two for each time frame, except Future, which was reflected by a single (mainly positive) scale. Past Positive captures a warm, positive, and nostalgic view of the past. Past Negative, by contrast, reflects a negative and aversive view of the past. The scales involving relations to the present time frame distinguish a live-for-the-moment attitude involving immediate pleasure seeking without much concern of future consequences labeled as Present Hedonistic and Present Fatalistic. Present Fatalistic reflects a hopeless and helpless attitude toward the present. Finally, the subscale called Future captures a broad future orientation that reflects optimism, planning, and striving for future rewards. An increasing number of studies also considered a measure known as Deviations from a Balanced Time Perspective (DBTP; Stolarski et al., 2011; Rönnlund et al., 2017) that takes into account deviations from a proposed ideal, or balanced, TP (Zimbardo and Boyd, 2008), across all of the ZTPI subscales. There is abundant empirical evidence supporting the notion that individual differences in TP as reflected by ZTPI are predictive of a broad set of behavioral and psychological outcome variables (see Stolarski et al., 2015; Kostic and Chadee, 2017).

Available evidence regarding the relationship between TP and decision making was to our knowledge restricted to few studies mainly in the area of propensity for risk-related decision (Jochemczyk et al., 2017) and career decision making (Walker and Tracey, 2012; Jung et al., 2015). Only more recently, TP has been investigated in relation to a more general aspect of decision making, but focusing on decision-making style, i.e., “… the learned, habitual response pattern exhibited by an individual when confronted with a decision situation” (Scott and Bruce, 1995, p. 820). Under the assumption that individuals may be characterized by their particular profile across a select number of styles, Scott and Bruce developed the General Decision Making Style questionnaire (GDMS). GDMS involves five styles: rational (systematic and extensive evaluation of options), intuitive (relying on hunches and emotions), spontaneous (impulsivity of decisions), dependent (to seek advice and support of others), and avoidant (to avoid decision whenever possible).

Carelli et al. (2011) showed several associations between the separate GDMS and TP dimensions. Specifically, a rational style was positively associated with scores on Future/Future Positive (*r* = 0.48/0.45), while the intuitive style was associated with higher scores on Past Positive, Present Fatalistic, and Present Hedonistic (*r* values: 0.33–0.35). The spontaneous style was related to higher Past Negative and Present Fatalistic. In turn, a dependent style was related to higher scores on Past Negative as well as Future Negative, as were scores on the scale reflecting the avoidant style. Similar associations between GDMS dimensions and TP dimensions were observed in a study involving an adolescent/student sample by Molinari et al. (2016). Thus, individuals scoring high on the negative temporal frame (Past/Future Negative) may tend to adopt a decision style that is avoidant and dependent. On the contrary, individuals who are more present focused tend to adopt a more intuitive and spontaneous decision style, while a rational style is associated with being more future oriented.

### Aims and Hypotheses of the Present Study

In the present study, we aim to extend the limited existing literature on the relation between aging, TP, and decision making by focusing specifically on decision-making competence. We start from the view that both a proper motivational approach to the task (Strough et al., 2015b) and an adequate cognitive ability (in particular working memory: Del Missier et al., 2013; Del Missier et al., 2017) are needed to achieve a good decision-making performance in cognitively challenging DMC tasks. Existing research showed that a Present Fatalistic TP is related to a less structured and analytic approach to decision making (Carelli et al., 2011; see also Baumann and Odum, 2012). This fact possibly reflects lower motivation individuals with a Present Fatatlistic TP as other studies linked this TP with lower motivation (e.g., Stanescu and Iorga, 2015; Zajenkowski et al., 2016a). For example, a strong negative correlation between Present Fatalistic and a measure of achievement motivation (Stanescu and Iorga, 2015) was observed. Finally, aging is associated both with increased Present Fatalistic time orientation (Rönnlund et al., 2017) and with decreased working memory performance (e.g., Park et al., 2002; Del Missier et al., 2013, 2017). Building on this knowledge, we expect that the age-related decrease in decision-making competence observed in previous studies (e.g., Bruine de Bruin et al., 2012; Del Missier et al., 2017; Rosi et al., 2019) will be been mediated both by age-related differences in Present Fatalistic time orientation, reflecting lower task-oriented motivation, and by age-related differences in working memory.

Therefore, our hypotheses for the present study were as follows: age will be negatively associated with DMC performance and with working memory performance, but positively associated with Present Fatalistic. Moreover, participants with a more Present Fatalistic TP will score lower on DMC tests (due to their less proactive and structured approach to the decision tasks) and participants with lower working memory performance will also show a lower DMC performance (due to the functional support of working memory to cognitively challenging DMC tasks). Finally, we expect that age-related differences in DMC will be at least partially mediated by age-related differences in both Present Fatalistic time orientation and working memory. For what concerns the relation between TP and working memory, we expect to find a negative association between Present Fatalistic and working memory, following up previous research showing relations between Present Fatalistic and various higher-order cognitive functions closely related to working memory, including general cognitive ability (Rönnlund and Carelli, 2018a), fluid intelligence (Zajenkowski et al., 2016a, b), and working memory updating (Witowska and Zajenkowski, 2019). To examine the possibility that other TP dimensions contribute to efficient DMC, we considered the other ZTPI [Swedish Zimbardo Time Perspective Inventory (S-ZTPI); Carelli et al., 2011] dimensions as well together with a measure capturing TP biases across all of the dimensions (DBTP; Stolarski et al., 2011; Rönnlund et al., 2017).

## Materials and Methods

### Participants

Our sample was composed of 346 Swedish adults ranging in age from 60 to 90 years (M = 70.2, SD = 4.4), including 138 adults aged 60–65 years, 145 aged 70–75, and 133 aged 75 or older. There were 167 males and 179 females in the sample. Informed consent was obtained from all participants and the study was approved by the regional ethical review board in Umeå.

### Procedure and Measures

Participants were screened for dementia, sensory mental retardation, sensory impairments, and a native tongue other than Swedish (for further details concerning sampling and inclusion, see Nilsson et al., 1997, 2004; Nyström et al., 2019). The participants underwent a health assessment session and a cognitive testing session in two different days, 1 week apart. A paper-and-pencil version of the ZTPI was given to the participants at the end of the health assessment. The questionnaire was completed at home and was returned at the cognitive testing session. Participants completed the Swedish version of the Adult Decision-Making Competence (A-DMC) tasks and other measures at home. They were provided with detailed written instructions and examples for each task, and they completed the A-DMC tasks alone and without external aids following the pre-specified task order.

#### Adult Decision-Making Competence

The A-DMC battery^1^ was developed to capture the skills central to normative theories of decision making (Parker and Fischhoff, 2005; Bruine de Bruine et al., 2007). The A-DMC originally included six scales. Data on four of the scales were collected in the present study: *Resistance to Framing*, *Recognizing Social Norms*, *Applying Decision Rules*, and *Resistance to Sunk Costs*. The scales *Under/Overconfidence* and *Consistency in Risk Perception* had not been included as part of the battery due to inconsistent findings in other studies in different countries in relation to their age-related changes and to the practical need to keep the data collection constrained. *Resistance to Framing* employs risky framing and attribute framing problems (Levin et al., 1998) to measure whether participants’ preferences are affected by normatively irrelevant variations in how options are described (e.g., ground beef described as either as “20% fat” or as “80% lean”). Fourteen item pairs are presented in two sets, with one containing the positive/gain member of each pair, and the other containing the corresponding negative/loss items. Performance is assessed with a score reflecting the participant’s ability to avoid being swayed by the superficial presentation of the problem (with higher scores associated to greater resistance to framing). *Recognizing Social Norms* asks “out of 100 people your age, how many would say it is sometimes OK” to engage in each of 16 undesirable behaviors (e.g., “steal under certain circumstances”). These perceived social norms are compared to the percent of respondents from the study who had reported earlier that “it is sometimes OK” to engage in each behavior (actual social norms of the group). Each participant’s score is the within-subject correlation between judged norms and observed norms. A higher score is associated to a better perception of social norms. *Applying Decision Rules* assesses the ability to apply specified decision rules (e.g., elimination by aspects, lexicographic) to 10 hypothetical choices, with each option characterized on several attributes in a table. Performance is assessed by the proportion of decision problems correctly solved via an errorless application of the prescribed decision rules. *Resistance to Sunk Costs* involves 10 sunk cost problems to assess the ability to ignore prior investments that are irrecoverable (sunk cost options) and consider only future consequences (better future options) when making decisions. A higher score in this task is associated with a greater resistance to sunk costs. Estimates of reliability for the components in studies using the Swedish version of the A-DMC (Del Missier et al., 2013) were as follows: 0.59 for Resistance to Framing, 0.83 for Applying Decision Rules, 0.78/0.90 (self/others) for Recognizing Social Norms, and 0.48 for Resistance to Sunk Costs.

#### Swedish Zimbardo Time Perspective Inventory

Swedish Zimbardo Time Perspective Inventory was used to assess TP. S-ZTPI (Carelli et al., 2011) consists of 64 items, each of which reflects one of six dimensions: Past Negative (e.g., “Painful past experiences keep being replayed in my mind”), Past Positive (e.g., “Familiar childhood sights, sounds, smells often bring back a flood of wonderful memories”), Present Fatalistic (e.g., “Fate determines much in my life”), Present Hedonistic (e.g., “I believe that getting together with one’s friends to party is one of life’s important pleasure”), Future Negative (e.g., “To think about my future makes me sad”), and Future Positive (e.g., When I want to achieve something, I set goals and consider specific means for reaching those goals”). The participant is requested to rate how characteristic each of the statements is of his/her own view on five-point Likert scale, ranging from *very uncharacteristic* (coded 1) to *very characteristic* (coded as 5). The S-ZTPI differs from the original in that it differentiates positive and negative aspects of the future TP by adding (eight) new items to inventory for the Future Negative scale. Future Negative reflects a broadly aversive view of the future. This distinction between Future Positive and Future Negative has been supported by several recent studies showing differential associations of the two future scales with perceived stress (Rönnlund et al., 2018), coping styles (Blomgren et al., 2016), well-being (Rönnlund et al., 2017), and sleep quality (Rönnlund and Carelli, 2018b). Confirmatory factor analyses provided support of the six-factor version and internal consistencies ranged from 0.65 to 0.94 across subscales (Carelli et al., 2011).

To capture TP biases across all the TP dimensions, we additionally considered the measure known as Deviations from a Balanced TP (DBTP) developed by Stolarski et al. (2011) and revised by Rönnlund et al. (2017; i.e., to take into account the Future Positive/Negative Distinction). DBTP was computed according to the formula (Stolarski et al., 2011; Rönnlund et al., 2017):

$$\sqrt{{\left(\mathrm{oPN}-\mathrm{ePN}\right)}^{2}+{\left(\mathrm{oPP}-\mathrm{ePP}\right)}^{2}+{\left(\mathrm{oPF}-\mathrm{ePF}\right)}^{2}+}$$

$${\left(\mathrm{oPH}-\mathrm{ePH}\right)}^{2}+{\left(\mathrm{oFP}-\mathrm{eFP}\right)}^{2}+{\left(\mathrm{oFN}-\mathrm{eFN}\right)}^{2},$$

where o = optimal score and e = empirical (i.e., observed) score. In accord with previous studies (Stolarski et al., 2011; Rönnlund et al., 2017), optimal scores were set to the following: oPN = 1.95, oPP = 4.6, oPF = 1.5, oPH = 3.9, oF/oFP = 4.0, and oFN = 1.8.

#### Working Memory

We employed two measures previously used as indicators of working memory in the Betula project (Del Missier et al., 2017). The first was the *2-back task*, a computerized version of the n-back paradigm used to assess the ability to update working memory contents (e.g., Owen et al., 2005). In this version of the task, 40 words are shown one after the other and the participants are required to keep in memory the most recent ones and their temporal order and to indicate whether the current word is the same as the one presented two items earlier or not by pressing two designated keys. The task is preceded by two rounds of 15 practice items. The performance score was the proportion of correct responses. The second measure used as a proxy for working memory was the WAIS-R Block Design test. This test involves the manipulation of cubes to match a series of patterns (provided on pictures) and was administered in accordance with the WAIS-R-manual (Wechsler, 1981). Raw scores were used as performance measure. Performance in this test proved to be strongly and selectively related to working memory updating (Friedman et al., 2006), consistently with other findings that showed a strong relation between working memory capacity and fluid intelligence (e.g., Carpenter et al., 1990; Engle et al., 1999; Kane et al., 2005). The 2-back reliability was 0.76 (Del Missier et al., 2017), while the Block design 5-year reliability was 0.81 (Rönnlund and Nilsson, 2006 – 5-year stability).

## Results

Descriptive statistics of the measures examined in the present study, including decision-making competence, TP, and working memory, are provided in Table 1. The intercorrelations of the measures of DMC, TP, and WM are presented in Table 2.

**TABLE 1:** Descriptive statistics of the measures included in the study.

**Measure**	***N***	***M***	***SD***	**Min**	**Max**
A-DMC – RTF	346	3.82	0.58	1.79	5.00
A-DMC – ADR	346	0.71	0.19	0.00	1.00
A-DMC – RSN	346	0.55	0.22	–0.40	0.94
A-DMC – RSC	346	4.62	0.66	2.50	6.00
S-ZTPI – Past Negative	346	2.25	0.59	1.10	4.30
S-ZTPI – Past Positive	346	3.57	0.52	1.33	5.00
S-ZTPI – Present Fatalistic	346	2.47	0.53	1.00	4.22
S-ZTPI – Present Hedonistic	346	2.87	0.45	1.56	4.53
S-ZTPI – Future Negative	346	2.49	0.55	1.11	4.20
S-ZTPI – Future Positive	346	3.28	0.43	1.82	4.55
DBTP	346	2.32	0.55	0.99	4.79
WM – Block Design	345	26.60	9.16	4	49
WM – 2-back	308	0.82	0.10	0.18	1.00

*A-DMC, Adult Decision Making Competence; RTF, Resistance to Framing; ADR, Applying Decision Rules; RSN, Recognizing Social Norms; RSC, Resistance to Sunk Costs; S-ZTPI, Swedish Zimbardo Time Perspective Inventory; DBTP, Deviations from a Balanced Time Perspective; WM, working memory.*

**TABLE 2:** Zero-order correlations of the measures in the study.

	**1**	**2**	**3**	**4**	**5**	**6**	**7**	**8**	**9**	**10**	**11**	**12**	**13**
(1) A-DMC – RTF	1												
(2) A-DMC – ADR	0.23^∗∗^	1											
(3) A-DMC – RSN	0.18^∗∗^	0.33^∗∗^	1										
(4) A-DMC – RSC	0.00	0.15^∗∗^	0.08	1									
(5) SZTPI – Past Negative	–0.07	–0.21^∗∗^	–0.05	0.00	1								
(6) SZTPI – Past Positive	–0.05	0.04	–0.08	0.04	–0.21^∗∗^	1							
(7) S-ZTPI – Pres Fatalistic	–0.07	–0.36^∗∗^	–0.18^∗∗^	–0.07	0.37^∗∗^	0.08	1						
(8) S-ZTPI – Pres Hedonistic	–0.02	–0.08	−0.13^∗^	0.03	0.17^∗∗^	0.27^∗∗^	0.38^∗∗^	1					
(9) S-ZTPI – Future Negative	0.00	–0.10	–0.03	–0.04	0.64^∗∗^	–0.06	0.40^∗∗^	0.15^∗∗^	1				
(10) S-ZTPI – Future Positive	0.02	0.10	0.00	0.04	0.18^∗∗^	0.13^∗^	–0.08	0.00	0.34^∗∗^	1			
(11) DBTP	–0.05	–0.29^∗∗^	–0.07	–0.09	0.55^∗∗^	–0.57^∗∗^	0.49^∗∗^	–0.22^∗∗^	0.45^∗∗^	–0.20^∗∗^	1		
(12) WM – Block Design	0.23^∗∗^	0.39^∗∗^	0.16^∗∗^	0.04	–0.16^∗∗^	0.02	–0.24^∗∗^	–0.02	–0.07	0.05	–0.20^∗∗^	1	
(13) WM – 2-back	0.12^∗^	0.26^∗∗^	0.16^∗∗^	0.01	−0.14^∗^	–0.04	–0.26^∗∗^	0.03	−0.11^∗^	–0.05	–0.19^∗∗^	0.41^∗∗^	1

*^∗^*p* < 0.05, ^∗∗^*p* < 0.01. A-DMC, Adult Decision-Making Competence; RTF, Resistance to Framing; ADR, Applying Decision Rules; RSN, Recognizing Social Norms; RSC, Resistance to Sunk Costs; S-ZTPI,Swedish Zimbardo Time Perspective Inventory; DBTP, Deviations from a Balanced Time Perspective; Pres, Present; WM, Working Memory.*

**FIGURE 1:**
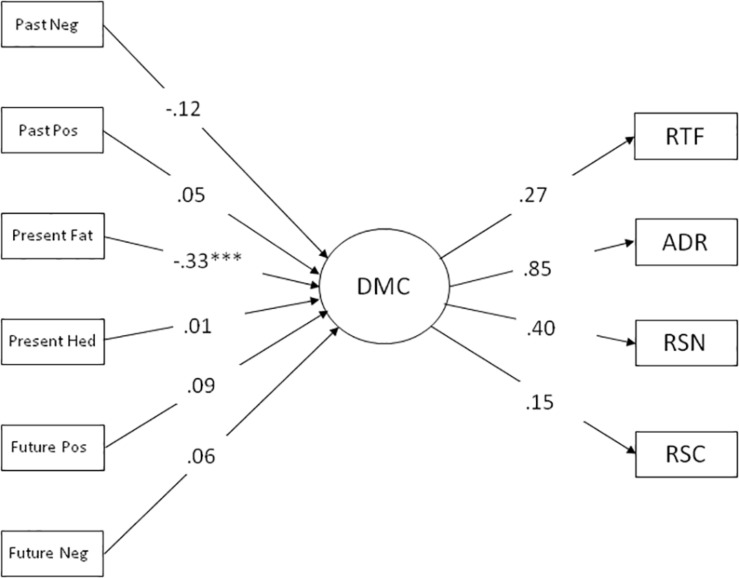
Structural equation model with the six S-ZTI dimension as the predictors of a latent DCM variable. Values are standardized coefficients. Pos, Positive; Neg, Negative; RTF, Resistance to Framing; ADR, Applying Decision Rules; RSN, Recognizing Social Norms; RSC, Resistance to Sunk Costs. ^∗∗∗^*p* < 0.001.

In the first step of the data analysis, we set out to examine the extent to which the TP dimensions were (uniquely) related to a general decision-making competence factor (Weller et al., 2018). Toward this aim, we tested a model where the four individual A-DMC components loaded on a single factor with the six TP dimensions as the predictors. Given prior demonstrations that some TP dimension showed significant correlations with age (*r* = 0.24, *p* < 0.001 for Present Fatalistic and *r* = 0.15, *p* < 0.01 for Future Negative in the present sample confirmed such associations) as was the measure of DBTP (*r* = 0.14, *p* = 0.01 in the present sample), age was controlled for in the analyses. The TP dimensions were allowed to correlate. Even though the strength of the loadings varied considerably (0.15–0.85; see also Weller et al., 2018), each of the A-DMC components showed significant loading on the DCM factor. The model showed good fit, χ^2^(23) = 23.96, χ^2^/df = 1.042, CFI = 0.998, RMSEA = 0.011. The basic model with values for paths/loadings is depicted in Figure 1.

Present Fatalistic was a highly significant predictor of DMC (β = -0.33, *p* < 0.001). None of the other S-ZTPI dimensions were uniquely associated with the latent DMC factor. Provided that Present Fatalistic was positively associated with age (*r* = -0.24) and negatively related to working memory measures (*r* = -0.24 and *r* = -0.26, with Block Design and 2-back, respectively), we next considered a model by which the expected negative effect of age on the DMC factor was mediated both by an age-related increase in Present Fatalistic and by an age-related decrease in working memory capacity.

In this model, each of the nine individual S-ZTPI items reflecting Present Fatalistic was included as separate indicators of a latent (Present Fatalistic) factor. The two cognitive measures (2-back, Block Design) served as indicators of a latent Working Memory factor. As in the previous analyses, the four DMC components were, finally, included as indicators of a latent DMC factor. Finally, the two cognitive measures (2-back, Block Design) served as indicators of a latent Working Memory factor.

**FIGURE 2:**
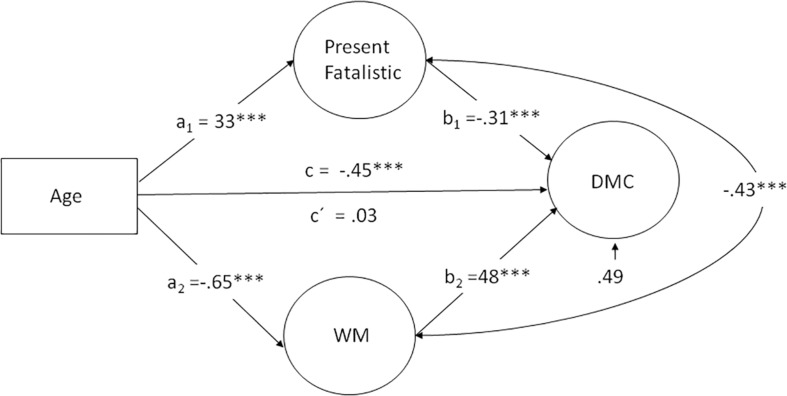
Relationship between age and latent-level decision making competence (DMC) variable with Present Fatalistic and working memory (WM) as mediators of the effect. a and b are direct effects. c is the total effect of age, c’ is the direct effect of age controlling for Present Fatalistic and working memory. ^∗∗∗^*p* < 0.001.

To obtain tests of indirect effects and to obtain bias-corrected (95%) confidence intervals (BCIs) for the estimates, we used bootstrapping, involving 1000 bootstrap samples. This method requires complete data. We therefore used a regression-based approach to impute missing values on the cognitive measures (see Table 1). The imputation procedure had virtually no effect on the β-values (analyses based on participants with complete data showed a highly similar result). A summary of the model and the results (β-values for the paths) is shown in Figure 2.

The model fit was good, χ^2^(99) = 183.37, χ^2^/df = 1.852, CFI = 0.903, RMSEA = 0.05. The results confirmed a substantial total effect of age on DMC (β = -0.45, 95% BCI: -0.57 to -0.33), and a significant indirect effect of age on DMC (β = -0.42, 95% BCI: -0.61 to -0.27) but no direct effect of age on DMC (β = -0.03, 95% BCI = -0.21 to 0.18) with a ratio of indirect to total effect of 0.93. The links from age to WM (β = -0.65), from age to Present Fatalistic (β = 0.33), and from WM and Present Fatalistic to DMC (-0.48, -0.31) were all highly significant. Thus, the results provided support for full mediation of the effects of age on DMC.

In order to examine the possibility that cohort differences in education (years of schooling) biased the foregoing estimates, a second model was run in which this factor was controlled for. Inclusion of this factor did not change the presence or absence of any of the foregoing effects supporting the robustness of the findings obtained. Finally, the fact that the loadings of the individual A-DMC components on the general DMC factor varied considerably may suggest that analyses should be performed at the level of separate A-DMC components. Such analyses revealed significant age associations (*p* < 0.05) for three of the individual A-DMC components: Applying Decision Rules (β = -0.36), Resistance to Framing (β = -0.12), and Recognizing Social Norms (β = -0.21), two of which were significantly related to Present Fatalistic as well as working memory (see Table 2). However, only for ADR was the pattern of full mediation of age effects together with a significant (unique) association with Present Fatalistic (cf. Figure 2) observed.

## Discussion

The present study examined relations between TP and decision-making competence in an older sample. The results confirmed a significant relationship between one of the TP dimensions, Present Fatalistic, and a general DMC factor. The selective relationship between this particular facet of TP and DMC is noteworthy, in that Present Fatalistic accounted for variance in a general DMC factor over and beyond working memory, which was already established as a major factor contributing to effective decision making (Del Missier et al., 2013, 2017) in particular for more demanding DMC components such as Applying Decision Rules. In total, the two factors accounted for a little more than half of the variance in DMC, which is rather substantial. Additionally, in agreement with our predictions based on studies involving related cognitive measures (e.g., Zajenkowski et al., 2016a, b; Rönnlund et al., 2018; Witowska and Zajenkowski, 2019), we observed a significant association between a latent working memory variable and a latent Present Fatalistic variable over and beyond age. More generally, the data were consistent with a model by which the negative age effect on a general DMC factor is mediated by age-related decreases in working memory and increased present fatalistic attitudes.

Theoretical frameworks posit that, in addition to effective cognitive processing, rational decision making requires an appropriate motivational orientation to the task (Strough et al., 2015b, see also Bruine de Bruine et al., 2015). It appears reasonable to consider motivational factors to account for the link between Present Fatalistic and DMC. Being focused on the present in the fatalistic sense should undermine motivation to carry out cognitively demanding tasks, as control of future consequences and outcomes of behavior are regarded as out of own control. This should entail lower levels of self-efficacy (see Boniwell et al., 2010; Zebardast et al., 2011; Zajenkowski et al., 2016a, b) and a less proactive and organized approach to the task (Carelli et al., 2011, see also Baumann and Odum, 2012). Lower self-efficacy in the decision-making competence domain, or more generally, could possibly hamper performance in cognitively demanding decision-making tasks. In a related vein, it could be that those who are more present fatalistic are more selective in regard to what tasks they invest effort, devaluing decision-making tasks lacking in terms of direct personal relevance, an age-related motivational constraint that has been suggested to contribute to poorer DMC in older adults more generally (Löckenhoff and Carstensen, 2007; Hess et al., 2015). Indeed, recent studies support the notion that age-related changes in motivation can affect the older adults’ effort to make decisions (Bruine de Bruine et al., 2015; Strough et al., 2015a). At this point, it is warranted to point out that even though we found support of a general DMC factor, the loadings of the individual tasks varied considerably (see also Weller et al., 2018) and the most cognitively demanding factor (Applying Decision Rules) with the highest loading on the latent facto was likely the main driver of the associations with age and Present Fatalistic.

Rönnlund et al. (2017) (see also Desmyter and De Raedt, 2012) found a clear age-related increase in Present Fatalistic, while Past Positive, Present Hedonistic, and Future Positive were relatively stable across age. A key concept in trying to explain the present fatalistic age bias in old age is “locus of control,” defined as individuals’ beliefs about how personal actions, chance, and powerful others determine life events and circumstances (Levenson, 1973). Individuals who attribute their life circumstances to external resources such as fate – as in present fatalistic TP – are unlikely to plan for the future (Shipp et al., 2009). Higher external locus of control, in contrast to “internal locus of control” – individuals who believe that their life circumstances result from their own behavior (Rotter, 1966) – is often reported in old age (e.g., Caplan and Schooler, 2003). This shift toward an external locus of control/Present Fatalistic in late senescence could, in part, stem from physical and cognitive impairments (Lachman, 1986; Rönnlund et al., 2017; Rönnlund and Carelli, 2018a). Hence, it is possible that lower levels of cognitive functioning are a forerunner of higher levels of Present Fatalistic. In particular, lower cognitive flexibility could make an individual more likely to “get stuck” in a particular TP bias, such as a Present Fatalistic TP bias (e.g., Zajenkowski et al., 2016b). However, a reversed causal influence (i.e., from locus of control/PF to cognitive task performance) is additionally worth considering (e.g., Zajenkowski et al., 2016a, b) and the present finding of a link between Present Fatalistic and DMC over and beyond variations in working memory ability indicates a need to consider such influences in the context of decision-making performance.

From a broader perspective, our findings seem consistent with the socio-emotional selectivity theory (SST, Carstensen et al., 1999; Carstensen, 2006), according to which the perception of time plays a fundamental role in the selection and pursuit of social goals, with important implications for emotion, cognition, and motivation. In old age, we tend to perceive our time horizons as limited and to be more present-time oriented, thus to prioritize personally relevant and proximal socio-emotional goals and related tasks (i.e., spending time with familiars and social partners).

### Limitations and Future Research Directions

Even though the present study had advantages that might be deserved to be highlighted, including comprehensive measures of the constructs, a reasonably large population-based sample, and strong reported effects, our investigation has its limitations. Of primary concern, the study design was cross-sectional. Even though tests of mediational models based on cross-sectional data can provide a check of the consistency of theoretical models, and even if we controlled for the potential role of education, longitudinal data are required to provide an evaluation of causal relationships among the variables. Additionally, future studies are needed to further clarify the relation between Present Fatalistic and motivational factors in determining DMC in older adults, given that we did not directly measure motivation to perform DMC tasks. In this context, it will be of interest also to see the extent to which particular devices, instructions, or training procedure can be used to minimize age differences in (demanding) aspects of DMC. The meta-cognition framework by Stolarski and Witowska (2017) seems to suggest that procedures aimed to increase temporal–metacognitive skills (or maintenance of such skills) in older adults might reduce TP biases, hence allowing for more efficient DMC. Future studies also needed to determine the extent to which the present associations generalize across age or whether the observed link between Present Fatalistic and DMC is particularly evident in older samples. Although our study provides a first significant contribution to the investigation of the relationships between age, time orientation, and cognitive abilities in decision-making competence, further research is needed to fully elucidate the intriguing network of relationships between these significant constructs that we started to unveil.

## Data Availability

The datasets generated for this study are available on request to the corresponding author.

## Ethics Statement

This study was approved by the Regional Ethics Review Board, Umeå, with informed consent from all subjects. All subjects gave written informed consent in accordance with the Declaration of Helsinki.

## Author Contributions

MR performed the data analyses and wrote the first draft of the manuscript. FD, TM, and MC made critical revisions of the manuscript.

## Conflict of Interest Statement

The authors declare that the research was conducted in the absence of any commercial or financial relationships that could be construed as a potential conflict of interest.

## Footnotes

1 http://www.sjdm.org/dmidi/Adult_-_Decision_Making_Competence.html
